# Extracellular vesicle-mediated crosstalk between pancreatic cancer and stromal cells in the tumor microenvironment

**DOI:** 10.1186/s12951-022-01382-0

**Published:** 2022-05-02

**Authors:** Ying Li, Wenjing Zhao, Yanli Wang, Haiyan Wang, Shanglong Liu

**Affiliations:** 1 Department of Blood Transfusion, the Affiliated Hospital of Qingdao University, Qingdao, China; 2 Central Laboratory, Peking Union Medical College Hospital, Chinese Academy of Medical Science & Peking Union Medical College, Beijing, China; 3 Department of Operating Room, The Affiliated Hospital of Qingdao University, Qingdao, China; 4 Department of Gastrointestinal Surgery, The Affiliated Hospital of Qingdao University, Qingdao, China

**Keywords:** Extracellular vesicles, Pancreatic cancer, Tumor microenvironment, Communication, Clinical applications

## Abstract

Pancreatic ductal adenocarcinoma (PDAC) interacts closely with the tumor microenvironment (TME). The TME is remodeled by crosstalk between pancreatic cancer cells and stromal cells, and is critical for cancer progression. Extracellular vesicles (EVs), including exosomes and microvesicles, help facilitate an exchange of information both within the TME and to distant organs. EVs have also been identified as potential diagnostic biomarkers, therapeutic targets, and drug carriers for pancreatic cancer treatment. Thus, understanding the selective packaging of EVs cargo and its mechanistic impact will increase our understanding of cancer biology. In this review, we collect and analyze recent findings of the pancreatic cancer-stromal cell interactions mediated by EVs and the mechanisms involved in cancer-related immunity and chemoresistance. These studies demonstrate the vital role of EVs in pancreatic cancer reprogramming and TME remodeling. We also summarize the EVs identified as potential PDAC diagnostic biomarkers and possible therapeutic targets. This greater understanding is a promising avenue for transitioning EVs from bench to bedside.

**Graphical Abstract:**

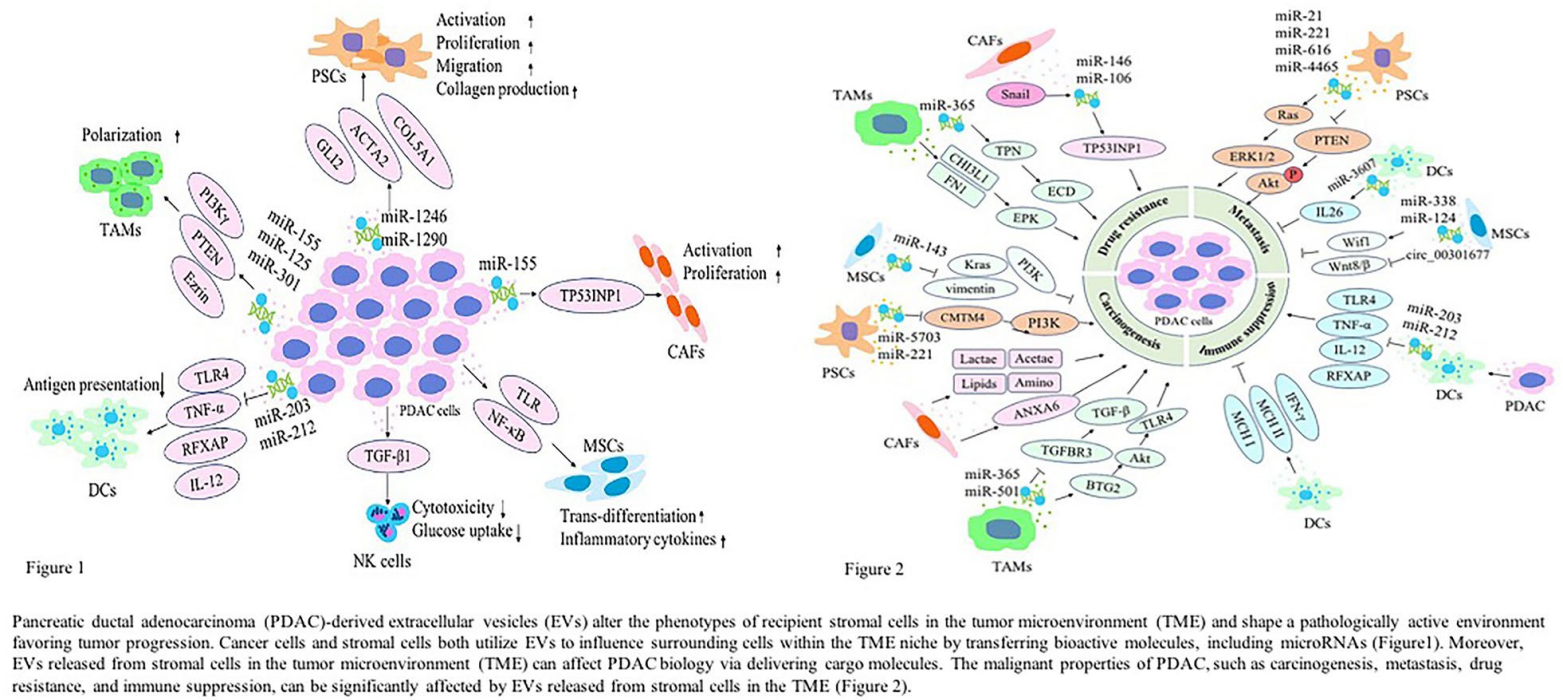

## Introduction

Pancreatic ductal adenocarcinoma (PDAC) is the fourth leading cause of cancer-related deaths and is expected to become the second leading cause of cancer-related deaths by 2030 [1]. The poor prognosis associated with the disease arises from late detection, aggressive tumor biology, and poor response to available therapies [2]. Current efforts predominantly focus on targeting cancer epithelial cell proliferation. In addition to tumor cells, the tumor microenvironment (TME) of PDAC is characterized by an exceedingly rich stroma that composes almost 90% of the tumor mass content [3]. Because the stroma has high solid stress, hypovascularity, and hypoperfused tumor vessels, it can restrict the therapeutic response to chemotherapy. Together with the extracellular matrix, a variety of cell types in the TME, including pancreatic stellate cells (PSCs), cancer associated fibroblasts (CAFs), endothelial cells, and immune cells, form a dynamic microenvironment that can promote cancer progression.

Extracellular vesicles (EVs) have emerged as important players in intercellular signaling and tumor-stroma interactions. Cells generate EVs to communicate with other cells at local or distant sites and modify the behavior of these target cells. EVs are encapsulated with a phospholipid membrane and can contain DNA, RNAs (microRNAs (miRNAs), mRNAs, and other small RNAs), lipids, proteins, and metabolites [4]. EVs play key roles in cell-to-cell communication under normal physiological conditions, including performing antigen presentation to directly activate CD4^+^ and CD8^+^ T cells and mediating bidirectional communication between the embryo and uterine endometrium during pregnancy, which is important for successful embryo implantation. EVs can have multiple roles in cancer, such as promoting cancer progression and priming sites for metastasis [5]. EVs from pancreatic cancer promote the recruitment and activation of stellate cells, while EVs from PSCs are involved in pancreatic cancer progression and promoting the secretion of EVs [6, 7]. Therefore, pancreatic cancer cells and PSCs form a positive feedback loop via EVs, demonstrating that EVs may play an important role in the development, growth, metastasis, and chemoresistance of a malignant tumor. However, the mechanism controlling how EVs can mediate tumor-stroma interactions and their role in TME remodeling and pancreatic cancer progression need further exploration. In this review, we will focus on the role of EVs in the communication between pancreatic cancer and stromal cells, and summarize the mechanism by which EV-mediated crosstalk can regulate pancreatic cancer progression. Finally, we will discuss the potential clinical applications of EVs as early diagnostic biomarkers and therapeutic modalities.

## Generation and properties of EVs in PDAC

### Morphology, biogenesis, and fusion of EVs

EVs are a heterogeneous population of cell-derived membrane vesicles with a wide size range. According to their sizes and biogenesis mechanisms, EVs are classified into three major groups: (1) exosomes, ranging from 30 to 150 nm in diameter, which are derived from the endosomal compartments; (2) microvesicles (MVs), which bud off from the plasma membrane and are large membrane vesicles of 150–1000 nm in diameter; (3) apoptotic bodies and oncosomes with 1000–5000 nm in diameter. Smaller shed MVs have also been reported, which are ~ 100 nm in diameter [8]. The lipid bilayer of EVs encapsulates a diverse array of bioactive cargo molecules and protects them from enzymatic degradation. Multiple mechanisms are involved in the biogenesis of EVs [8, 9]. Research has demonstrated that the CD44v6 and tetraspanin 8 (Tspan8) axis is involved in EV formation in pancreatic cancer [10]. CD44v6, mainly expressed in epithelial tumors, is an adhesive molecule that mediates interactions between cells and the extracellular matrix via its link domain and the HA binding site. It is essential for the invasive and metastatic potential of some tumors. It also supports stroma formation and assists premetastatic niche preparation by HA and matrix-remodeling enzymes. Günthert et al. reported that CD44v6 conferred metastatic potential to pancreatic carcinoma cells using in vivo experiments [11]. The metastasis-inducing Tspan8 is localized to the glycolipid-enriched membrane domains, which are prone to internalization throughout EV biogenesis. Tspan8 is a tetraspanin with an extracellular loop that mediates protein–protein interactions. Tspan8 can strengthen CD44v6, integrin, and cldn7palm/EpCAM complex signaling activity via its association with PKC and PI4K, thus promoting tumor progression. Moreover, the association with CD44v6 and/or Tspan8 can activate CXCR4 by enhancing its binding to ligand SDF1, which is important in PSC activation and the recruitment of immunosuppressive MDSCs and Tregs [10]. Other proteins are involved in EV biogenesis, including members of the integrin pathway, G-protein-coupled receptors (GPCRs), cytokines, and members of the notch signaling cascade, which can associate with Tspan8 and CD44v6 [12]. In addition, hypoxia has been shown to promote EV release via HIF-dependent expression of Ras-related protein Rab-22A [13]. Exosomes originate as intraluminal vesicles via inward budding of the maturing endosome membrane. The biogenesis of exosomes is distinct from that of EVs. One of the mechanisms involves the recruitment of the endosomal sorting complex required for transport (ESCRT) machinery to ubiquitinated proteins in the endosome [14]. There is also an ESCRT-independent exosome biogenesis pathway, which is mediated by the sphingolipid ceramide [15]. Some other key regulators of EV secretion and fusion with the plasma membrane include Rab family members (e.g., Rab27a/b, Rab7, Rab11 and Rab35), synaptotagmin-7, and tetraspanin CD63 in an ESCRT-independent mechanism [16, 17]. Certain physiological conditions in tumors, for example, hypoxia, low pH, and migratory behavior, can affect cancer cell-derived EV biogenesis. To summarize, the various mechanisms underlying the release of EVs can differ based on the types of host cells, its environment, and the content of the released EVs.

After being released from donor cells, EVs can fuse with the plasma membrane of target cells. During this process, EVs transfer biologically active cargo to recipient cells, thereby altering the properties of those cells. There are multiple processes by which EVs and their cargo molecules can be transferred to recipient cells, including (i) EVs attaching to the cells and acting as a ligand to activate receptors on the cell surface; (ii) EVs activating a receptor on the surface of the recipient cell without being taken up by the cell, (iii) EVs attaching to the cells and fusing, or (iv) the entire EVs is taken up by endocytosis [18, 19]. Once released, the fate of the EVs is based on their size, composition, and the microenvironment. In pancreatic cancer, EVs are secreted by three main cell types: cancer cells, stromal cells (e.g., CAFs, PSCs), and immune cells (i.e., T and B cells, NK cells, and macrophages) [20, 21]. EVs derived from different cells in the intracellular communication process can coordinate cell behaviors in numerous ways. However, the further fate of the EVs inside the cells remains unclear, and the process by which EVs release their content in the recipient cell cytoplasm needs further investigation.

### EV cargo in pancreatic cancer

There are three EV databases that provide detailed information about the molecules inside EVs: ExoCarta (http://www.exocarta.org), EVpedia (http://evpedia.info), and Vesiclepedia (http://www.microvesicles.org). EVs contain numerous molecules, including proteins, lipids, metabolites, mRNA, mitochondrial DNA, miRNAs, and many other non-coding RNAs. EVs are heterogeneous in their size and cargo, even when they are derived from the same cell.

### Proteins

EVs not only contain specific proteins that reflect the cell of origin, but also a specific subset of proteins that are shared between different cell types. The shared proteins are involved in EV biogenesis and fusion, while the unique proteins reflect the cell of origin and thus have been proposed as biomarkers. Tetraspanin and integrin proteins are essential for cell targeting and adhesion, while Rab GTPases, annexins, and flotillins are crucial for EV fusion. Heat shock proteins (HSP)70 and HSP90 are molecular chaperones and also involved in EV biogenesis [22, 23]. Characterization of the proteomes of human pancreatic cancer and non-malignant human pancreatic epithelial cell line-derived EVs indicated that 362 proteins are specifically expressed. Oncogenic EVs contain factors known to regulate the pre-metastatic niche (NCSTN, S100A4, F3, ITGβ5, ANXA1), clinically relevant proteins that are associated with prognosis (CLDN1, MUC1), as well as proteins involved in cancer progression, including proliferation (CLU, CAV1), invasion (PODXL, ITGA3), metastasis (LAMP1, ST14), and immune surveillance escape (B2M) [24]. The protein packaging between cancers of different origins has specific metastatic organ-tropisms. $\alpha \nu \beta$ 5 integrin was abundant in EVs from pancreatic cancers that metastasize to the liver. Moreover, the integrin expression levels are higher in EVs derived from metastatic cells compared with the expression levels in the cells themselves [25].

### Nucleic acids

Many studies have confirmed the presence of mRNAs, non-coding RNAs, and miRNAs in EVs after Valadi and colleagues first reported in 2007 that EVs derived from mast cells contained mRNA and miRNA in [26]. MiRNAs appear to be an important molecular cargo and potential cancer biomarkers. They have diverse regulatory roles once taken up by the recipient cells, which include distinct cells in the tumor microenvironment. Specifically, miRNAs that have been identified as potential biomarkers for PDAC diagnosis and prognosis include miR-21, miR-17- 5p, miR-23b-3p, miR-191, and miR-451 [27–29].

Double-stranded DNA (dsDNA) is located on the surface and inside of the vesicle [30]. As one of the most stable EV cargoes, cytoplasmic DNA fragments can originate from either the nucleus or mitochondria. DNA damaged from ageing, aberrant DNA leakage, and oxidative stress can accumulate in the cytoplasm of tumor cells, which activates two of the main components of DNA sensor machinery including stimulator of interferon genes (STING) and cyclic GMP–AMP synthase (cGAS) proteins [31]. The STING pathway is indispensable in tumor treatment, as activation of STING has a synergizing effect on therapy in pancreatic cancer, whereas knockdown of STING or cGAS signaling is sufficient to abolish the efficacy of immune checkpoint inhibitors [32, 33]. EVs containing tumor-specific DNA mutations have diagnostic potential, with the possibility of performing “liquid biopsy” in patients [34]. Liquid biopsy, known as non-invasive sampling of bodily fluids, has been one of the most exciting breakthroughs for cancer diagnosis. Moreover, liquid biopsies provide a non-invasive avenue for molecular stratification and treatment monitoring. The biomarkers for liquid biopsy include circulating tumor cells (CTCs), circulating tumor DNA (ctDNA), and EVs. These three liquid biopsy biomarkers have both advantages and limitations [35]. Combining analysis of all these liquid biopsy biomarkers is feasible for effective cancer diagnosis and management because of the heterogeneity in biomarker levels within and among different individuals [36]. More investigation into combining multiple liquid biopsy biomarkers for PDAC diagnosis is needed to assess reproducibility, repeatability, feasibility, and cost effectiveness. DNA in EVs is likely to be more stable compared with ctDNA because of protection given by the lipid bilayer. Therefore, as a raw material for liquid biopsy, EVs could be superior over ctDNA alone [37]. Exosomal KRAS mutations are present in the early stages of PDAC, as well as during its advanced metastatic stage, indicating that circulating mutations in EVs could be used to develop early cancer diagnostic tools [38]. Interestingly, serum EV analysis of pancreatic cancer patients showed that the presence of KRAS-specific mutations can provide unique information on patient outcome and cancer progression [39]. Although EVs seem to be ideal biomarkers for research, some technical challenges persist, such as effective isolation, purification, and identification of specific EV populations. Utilizing EVs in liquid biopsy is still in the primary stage. Nonetheless, EV DNA-based liquid biopsy tests are likely to improve personalized therapy and patient outcomes.

### Lipids and metabolites

Compared with the cell plasma membrane, EVs are bound by a bilayer that contains various lipids, including ceramide, sphingomyelin, phosphatidylcholine, diacylglycerol, and gangliosides [40]. The lipid composition of EVs from PDAC is reportedly abundant in cholesterol and sphingomyelin, but depleted in phosphatidylserine [41]. Lysophosphatidylcholine, phosphatidylcholine, and phosphatidyl-ethanolamine in PDAC-derived EVs are associated with tumor stage, CA19-9, and CA242. Moreover, phosphatidyl-ethanolamine is also significantly correlated with patient overall survival [42]. Another study showed that Synthetic Exosome-Like Nanoparticles (SELN), composed of lipids similar to those found in EVs, could induce activation of NF-κB in pancreatic cancer cells and induce expression of the chemokine SDF-1α. This can then interact with CXCR4 on the surface of cancer cells and induce drug resistance via Akt signaling [43]. These results suggest that lipids in EVs may play a role in mediating drug resistance.

## EV-mediated communication in PDAC

### Crosstalk between PDAC and stromal cells in the TME

**Fig. 1:**
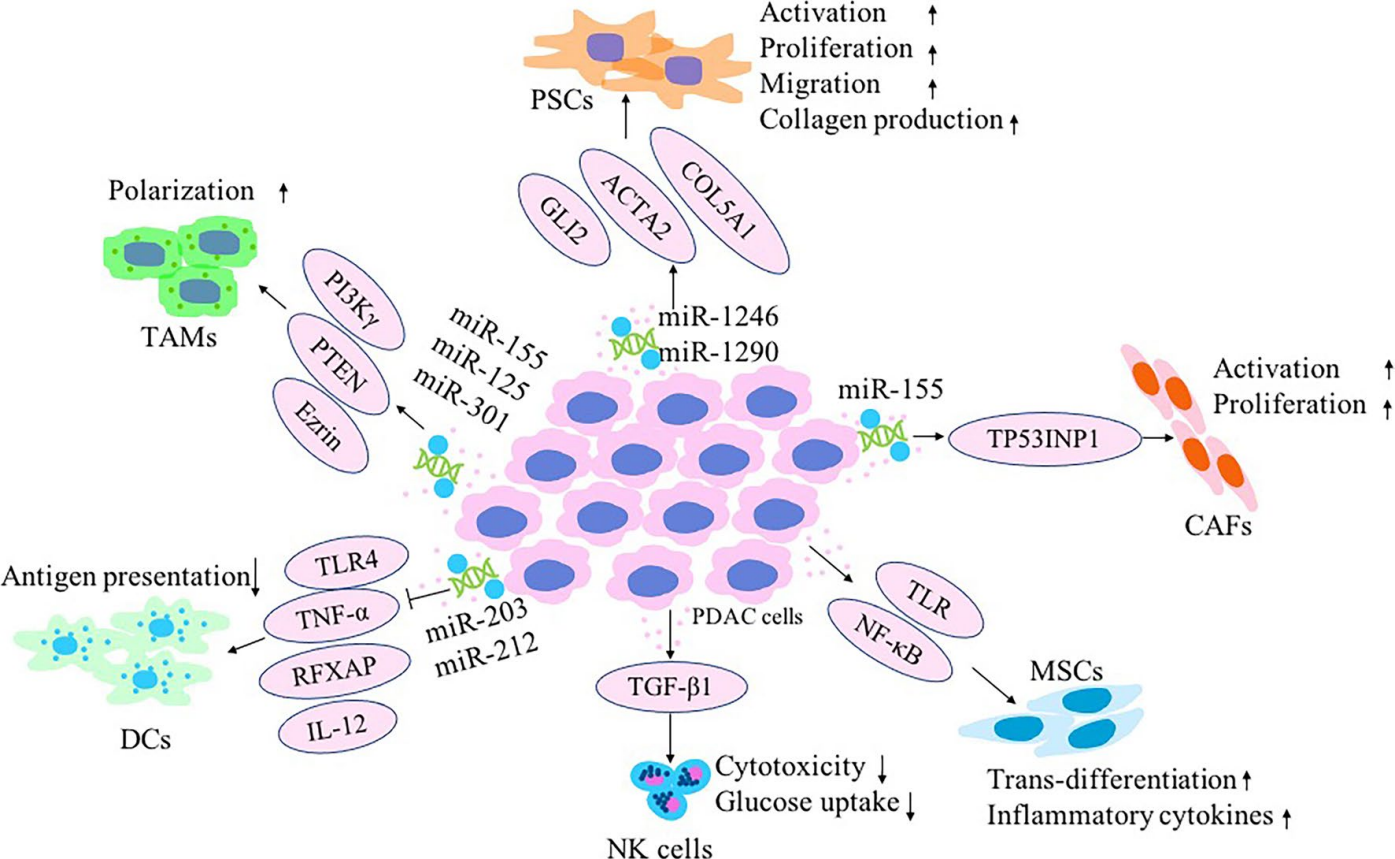
Multiple roles of pancreatic ductal adenocarcinoma (PDAC)-derived extracellular vesicles (EVs) in altering the phenotypes of recipient stromal cells in the tumor microenvironment (TME) and shaping a pathologically active environment favoring tumor progression. Cancer cells and stromal cells both utilize EVs to influence surrounding cells within the TME niche by transferring bioactive molecules, including microRNAs

**Fig. 2:**
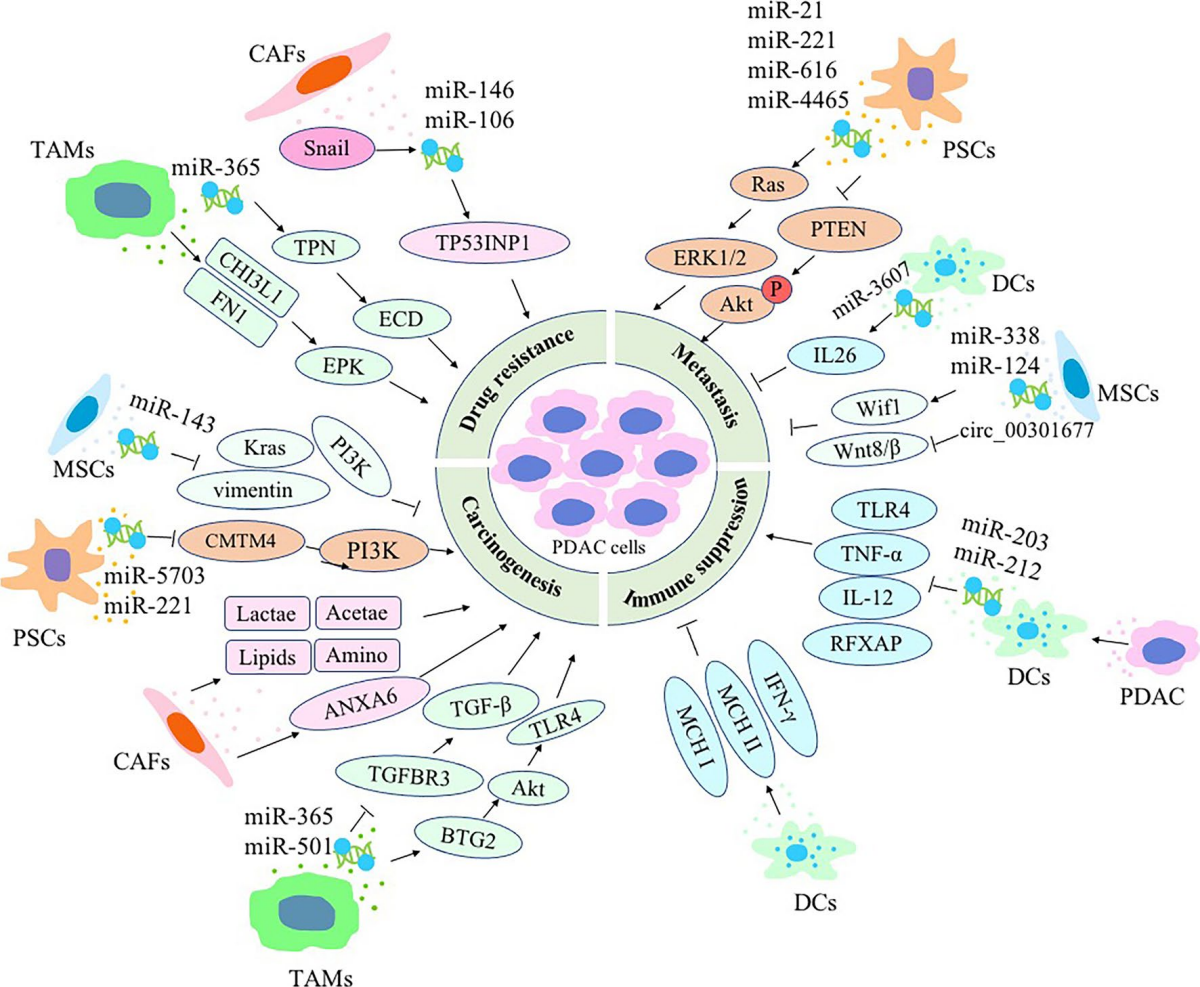
Extracellular vesicles (EVs) released from stromal cells in the tumor microenvironment (TME) can affect pancreatic ductal adenocarcinoma (PDAC) biology via delivering molecular cargo molecules. The malignant properties of PDAC, such as carcinogenesis, metastasis, drug resistance, and immune suppression, can be significantly affected by EVs released from stromal cells in the TME

Within the PDAC TME, quiescent PSCs are activated into CAFs. A number of immune cells, including myeloid-derived suppressor cells and tumor-associated macrophages (TAMs), infiltrate the TME and induce tumor immune tolerance. The PDAC TME also demonstrates a paucity of dendritic cells (DC) and natural killer (NK) cells [44]. The complex interactions between cancer cells and other cell types can occur either intercellularly or extracellularly, mediated by direct contact between cells or by the transfer of secreted molecules or EVs [45]. Recently, EVs were found to be efficient intercellular communication mediators that can regulate multiple cellular processes. Cancer-derived EVs can reshape the phenotype of macrophages and fibroblasts to promote cancer progression, thus rendering them a characteristic of TAMs and CAFs, respectively (Fig. 1) [46, 47]. Likewise, EVs from TAMs and CAFs have been demonstrated to promote cancer progression, metastasis, drug resistance, and immune evasion (Fig. 2) [48]. The key stromal cell types include PSCs, CAFs, TAMs, DCs, NK cells, and mesenchymal stem cells (MSCs) (Table 1). Here, we will discuss EVs released from tumors and various stromal cells while noting the interactions between PDAC and stromal cells in the TME.

**Table1:** Extracellular vesicles as message carriers between pancreatic cancer and stromal cells in tumor microenvironment

EVs source	Target cell	Cargo/Ligand Moiety	Key finding	Refs.
PDAC	PSCs	miR-1246 and miR-1290	Stimulate activation and profibrogenic activities in PSCs via activation of ERK and Akt	[57]
PDAC	CAFs	miR-155	Convert normal fibroblasts into CAFs via downregulation of TP53INP1	[71]
PDAC	TAMs	miR-155 and miR-125b-2	MiR-155 & miR-125b transfected PDAC-derived EVs polarized macrophages to M1-like phenotype	[79]
PDAC	TAMs	miR-301a-3p	Induce the M2 polarization of macrophages	[83]
PDAC	Non-polarized macrophages	ICAM-1 and arachidonic acid (AA)	Promote fusion via ICAM-1 and AA carried by exosomes and trigger macrophages to produce pro-tumorigenic factors VEGF, MCP-1, IL-6, IL-1β, MMP-9 and TNF-α	[84]
PDAC	TAMs	Ezrin	Polarize macrophages into a pro-tumor and have a significant impact on the survival of PDAC	[85]
PDAC	DCs	miR-203	Prevent DCs antigen presentation by downregulating TLR4	[97]
PDAC	DCs	miRNA-212-3p	Make DCs unable to activate CD4^+^ T cells	[98]
PDAC	DC	EVs as vaccine together GEM or Sunitinib	Reduce the activation and maturation of MDSC Suppress tumor cell migration and metastasis	[101]
PDAC	NK	TGF-β1	Suppress NK cell functions and reduce glucose uptake ability	[110]
PDAC	Kupffer cells	MIF	Promote fibronectin secretion and metastasis	[137]
PDAC	Stroma cell	CD151, Tspan8	Promote ECM degradation, reprogram stroma and hematopoietic cells	
Engineered PDAC	PDAC resistant to GEM	siRNA for RAB27B mRNA	Decrease exosome secretion and enhanced caspase-3/7 activity leading to apoptosis in GEM-resistant cancer cells	[16]
CAF	PDAC	Lactate, acetate, amino acids, lipids, and TCA cycle intermediates	Reprogram the metabolic machinery and promote tumor growth under nutrient stressed conditions in cancer cells	[60]
CAFs	PDAC	Snail (SNAI1) and its target, microRNA-146a	Promote cancer cells proliferation and gemcitabine resistance	[66]
CAFs	PDAC	miR-106b	Inhibit TP53INP1 gene and increase gemcitabine resistance	[67]
CAFs	PDAC	ANXA6/LRP1/TSP1	Increase aggressiveness	[68]
PSCs	PDAC	miR-5703	Downregulate CMTM4 and activate PI3K/Akt pathway to promote cancer cell proliferation	[49]
PSCs	PDAC	miR‑21	Promote PDAC cell migration and EMT and enhance Ras/ERK signaling activity	[52]
PSCs	PDAC	miR-22	Increase expression of K-Ras and NF-κB and contribute to desmoplastic reaction	[55]
PSCs	PDAC	miR-4465 and miR-616-3p	Promote proliferation and invasion via PTEN repression and AKT activation	[56]
TAMs	PDAC	miR-501-3p	Accelerate the development of PDAC via the activation of the TGF-β	[88]
TAMs	PDAC	miR-365	Enhance the proliferating, migrating and invading potentials of PDAC cells through BTG2/FAK/AKT axis	[89]
TAMs	PDAC	Toll-like receptor 4 (TLR4)	Promote EMT and aggressive behavior in pancreatic cancer cells	[90]
TAMs	PDAC	miR-365	Decrease the sensitivity of PDAC cells to gemcitabine	[91]
TAMs	PDAC	chitinase 3-like-1 (CHI3L1) and fibronectin	Induce PDAC resistance to gemcitabine through extracellular-signal-regulated kinase (ERK) activation	[92]
TAMs	Pancreatic cancer stem cells	miR-21a-5p	Mediate the differentiation and activities of pancreatic cancer stem cells via targeting krüppel-Like Factor 3 (KLF3)	[93]
NK	PDAC	miR-3607-3p	Suppress cancer cells migration and invasion through downregulation of IL-26	[109]
MSCs	PDAC	hsa-miR-143	Promote apoptosis and suppress cell growth, invasion, and migration	[118]
MSCs	PDAC	circ_0030167	Inhibit the invasion, migration, proliferation and stemness of pancreatic cancer cells	[119]
Pancreatic cancer stem cell	Gemcitabine-sensitive pancreatic cells	miR-210	Slow down cell cycle arrest at the G2/M phase, activates mTOR pathway, and induce gemcitabine resistance	[150]

### Pancreatic stellate cells (PSCs)

The normal functions of PSCs in the healthy pancreas include immune reaction, phagocytosis, and stimulation of amylase secretion. Any physiological or pathological reaction will activate PSCs. Activated PSCs promote the progression of pancreatic cancer through crosstalk with pancreatic cancer cells [49, 50]. The mechanisms controlling these interactions remain to be clarified.

Researchers have analyzed the EVs produced by PSCs to elucidate their composition and assess their role in PDAC. Among the various EV cargo molecules, miRNAs are recognized as central regulators of chromatin modification and gene regulation. Evidence shows that miRNAs may regulate perhaps more than 90% of the genome and affect relevant cellular processes [51]. Dysregulated miRNAs have been observed in many cancer types, including pancreatic cancer. Takikawa et al. described a microarray-based analysis of the miRNA signature in cultured human PSC-derived exosomes, which contained a variety of miRNAs such as miR-21-5p [52]. Upregulation of miR-21 (or miR-21-5p) can promote cancer progression by regulating the expression of numerous target genes, including the tumor suppressors PTEN, MSH2, Cdc25A, SPRY2, and PDCD4. PSC‑derived exosomal miR‑21 can promote PDAC cell migration and the epithelial‑to‑mesenchymal transition (EMT), and also enhance Ras/ERK signaling activity [53]. Several miRNAs, such as miR-451a, are enriched in EVs compared with the parental cells. MiR-451a regulates the drug-transporter protein P-glycoprotein, potentially increasing tumor resistance to the chemotherapy drugs [54]. Ali et al. identified PSC-derived exosomal miR-221 as a key mediator of PDAC cell progression [55]. Another PSC-derived exosomal miRNA, miR-5703, was found to promote the proliferation of PDAC cells by downregulating CMTM4 and activating the PI3K/Akt pathway [49]. Hypoxia can upregulate the expression levels of miR-4465 and miR-616-3p in EVs released from PSCs. These miRNAs can be transferred to pancreatic cancer cells, promoting proliferation and invasion via repressing PTEN and activating AKT [56].

Pancreatic cancer tumors often produce abundant amounts of exosomes. The cancer-derived exosomes can induce the activation and profibrogenic activities of PSCs, including proliferation, migration, collagen production, and $\alpha$-SMA (ACTA2) mRNA expression. Increased expression levels of miR-1246 and miR-1290 in pancreatic cancer-derived exosomes induces the expression of profibrogenic gene in PSCs [57]. Li et al. reported that PDAC-derived IL-17B-carrying EVs increased the expression of the IL-17B receptor on the surface of PSCs. They also induced IL-17RB expression, resulting in increased mitochondrial activity through enhanced oxidative phosphorylation. IL-17B/RB-signaling supplies energy for PDAC by increased oxidative phosphorylation and decreased glycolysis [58]. Inhibiting the crosstalk between these cancer cells and PSCs by blocking IL-17B/RB could be a potential targeted therapeutic approach for pancreatic cancer.

### Cancer-associated fibroblasts (CAFs)

CAFs develop from bone marrow-derived MSCs, PSCs, and quiescent resident fibroblasts. Sonic hedgehog (SHH), transforming growth factor-β (TGF-β), TNF-α, epidermal growth factor (EGF), platelet-derived growth factor (PDGF), fibroblast growth factor 2 (FGF2), and reactive oxygen species (ROS) are pivotal regulators of fibroblast activation [59]. Various markers for CAFs include stromal cell-derived factor-1α (SDF-1α), fibroblast activation protein (FAP), and fibroblast-specific protein-1 (FSP-1) [60]. Multiple pathways are involved in PDAC-CAF crosstalk. In addition to autocrine and paracrine signaling mediated by cytokines, mechanisms of CAF-tumor cell interactions also include EV transfer. Our previous study showed that there existed metabolism symbiosis between pancreatic cancer and stromal cells in TME [61]. Cancer cells can reshape CAFs metabolic features involved in their pro-tumoral effects via mitochondrial processing. CAFs in pancreatic cancer undergo metabolic changes from oxidative phosphorylation to aerobic glycolysis, thus producing metabolic intermediates including lactic acid and ketone bodies, and these metabolic intermediates can be directly taken up by tumor cells [62]. After metabolic reprogramming, the content of anaerobic metabolism related enzymes such as critical rate-limiting enzyme PKM2 in anaerobic metabolism increase significantly. Monocarboxylate transporter-4 (MCT-4) expressed on CAFs which is induced by HIF-1 mainly exports lactate from cells with glycolysis to prevent the intracellular accumulation of lactate. It promotes glucose transporter Glut1, lactate production, and extrusion of lactate, while MCT1 expressed on tumors increases to uptake lactate [63]. Glutamate secreted by cancer cells induces the glutathione pathway in CAFs, thus limiting the accumulation of ROS and superoxide and promoting the expression of glutamine synthase. Glutamine can reduce the autophagy of mitochondria, upregulate the expression of glutamine transporters, and enhance mitochondrial biosynthesis in pancreatic cancer [64]. Metabolic substrates can be transferred from CAFs to tumor cells via EVs. These EVs can contain lactate, acetate, amino acids, lipids, and tricarboxylic acid (TCA) cycle intermediates that are robustly utilized by cancer cells for central carbon metabolism and promoting tumor growth under nutrient deprivation or nutrient stressed conditions. Moreover, it is reported that EVs derived from breast cancer cells containing high level of miR-122 inhibited the glucose uptake by stromal cells via downregulating glycolytic enzymes such as pyruvate kinase, allowing cancer cells to preferentially take up glucose [65]. CAF-derived EV uptake by pancreatic cancer cells is inhibited by endocytosis inhibitor CytoD and receptor-mediated endocytosis inhibitor heparin independently of activated Kras expression levels [66]. CAFs are innately chemoresistant and have an active role in regulating the chemoresistance of cancer cells. CAFs exposed to gemcitabine can increase EV release, promoting cell proliferation and survival in recipient pancreatic cancer cells. Richards et al. found that the expression levels of Snail (SNAI1), as well as the Snail target, miR-146a, were increased in the EVs of CAFs exposed to gemcitabine. This promoted pancreatic cancer cell proliferation and drug resistance. Treatment of CAFs with GW4869, an inhibitor of EV release, significantly reduces survival of gemcitabine-resistant cancer cells [67]. The functions of miRNAs from CAF-derived EVs in regulating drug resistance have also been investigated. Fang et al. reported that miR-106b levels were upregulated in exosomes from CAFs following gemcitabine treatment, and this miRNA was directly transferred from CAFs to pancreatic cancer cells through the exosomes. MiR-106b promoted gemcitabine resistance of cancer cells by targeting TP53INP1. Pretreatment of CAFs with a miR-106b inhibitor resulted in a decreased degree of gemcitabine resistance in these cancer cells [68]. CAFs under physiopathologic culture conditions in the pancreatic cancer TME exhibit a protein complex involving ANXA6, LRP1, and TSP1. This complex can be exported onto EVs secreted by CAFs. Pancreatic cancer cells uptake these EVs to enhance their aggressive potential [69].

Pancreatic cancer cells can induce the conversion of normal fibroblasts to CAFs and, reciprocally, CAFs can promote tumor invasion and proliferation via EV-mediated crosstalk [67, 70]. Pancreatic cancer cells secrete EVs containing miR-155 to impact tumor-adjacent normal fibroblasts and convert them into CAFs [71]. TP53INP1 expression is repressed by miR-155 in pancreatic cancer, and its restoration inhibits pancreatic tumor development. The downregulation of TP53INP1 induced by MV-enclosed miR-155 from pancreatic cancer cells mediates the proliferation and activation of normal fibroblasts, and also manifests CAF characteristics [72]. Although some studies have demonstrated possible anti-tumor effects by CAFs, there is a lack of evidence supporting any anti-tumor effects on PDAC by CAF-EVs [73–75].

### Tumor-associated macrophages (TAMs)

TAMs, derived from the myeloid progenitor cells, are the most abundant infiltrative immune-related stromal cells present in the pancreatic cancer TME. Activated macrophages can be divided into classically (M1) or alternatively (M2) activated cells. M1 macrophages, characterized by the expression of the inducible type of nitric oxide synthase (iNOS), have pro-inflammatory and anti-tumor functions, whereas M2 macrophages express high levels of anti-inflammatory cytokines (e.g., IL-10) and have potent arginase-1 (Arg1) activity to promote tumor cell growth [76]. The myeloid cells that infiltrate into the tumor TME usually differentiate into TAMs and display the M2 phenotype. TAMs not only manipulate cancer cells toward growth and metastasis, but can also induce immune tolerance and chemoresistance. TAMs and M2 macrophages show similar functions, as both can respond to interleukin 4 (IL-4), IL-10, TGF-β, and IL-13, as well as promote tissue growth [77].

PDAC cells treated with miRNA-encoding plasmid DNA will show effects to the EV content and a change in macrophage polarization. Polarized macrophages in turn affect the biological behavior of PDAC cells [78]. Modifying the cargo inside the EVs using hyaluronic acid-poly (ethylene imine)-based nanoparticle delivery system (HA-PEI/HA-PEG)-encapsulated plasmid DNA expressing miR-155 or miR-125b-2 can achieve stable expression of these miRNAs [79]. MiR-155 has a critical role in macrophage polarization, as knocking it down resulted in the transitioning of macrophages to a M2/Th2 response [80]. MiR-125b is aberrantly expressed in tumors [81]. This miRNA induces surface activation markers in response to IFN-γ, which is overexpressed in macrophages. MiR-125b is enriched in M1 phenotype macrophages and has been associated with improved antigen presentation, enhanced T-cell activation, and tumor destruction [82]. The delivery system derived from pancreatic cancer can carry miR-155 and miR-125b, which can rehabilitate M2 macrophages back to the M1 phenotype. Moreover, PDAC cells generate miR-301a-3p-rich exosomes in a hypoxic microenvironment and induce the M2 polarization of macrophages via activation of the PTEN/PI3Kγ signaling pathway. Co-culturing of pancreatic cancer cells with macrophages in which miR-301a-3p is upregulated or treated with hypoxic exosomes could enhance their metastatic capacity [83]. EVs derived from pancreatic cancer cell lines incubated with non-polarized macrophages (THP-1 cells) caused higher expression of surface proteins CD14, CD163, and CD206, as well as higher secretion of pro-tumorigenic factors like VEGF, MCP-1, IL-6, IL-1β, MMP-9, and TNF-α. This can induce an M2-like phenotype in THP-1 cells [84]. Pancreatic cancer cells secrete Ezrin-rich EVs into the tumor microenvironment, which in turn polarizes macrophages that promote tumor metastasis [85]. Ezrin is a member of the Ezrin-radixin-moesin (ERM) family and regulates cell proliferation, migration, and adhesion, and also modulates plasma membrane signal transduction [86].

Effective strategies of TAM-targeted immunotherapy for cancer treatment includes repolarization of M2-like TAMs to the antitumorigenic M1-phenotype, decreasing the recruitment of infiltrating macrophages or inhibiting TAM survival. Inducing or repolarizing TAMs towards the M1-like phenotype in the tumor stroma is the most straightforward method. By employing antisense miRNAs (e.g., miR-155 and miR-125b) to inhibit M2-related pathways, the re-polarization of M2-like TAMs to the M1 phenotype can be achieved [87]. M2 macrophage-derived EVs can also be absorbed by pancreatic cancer cells. M2 macrophages deliver miR-501-3p through exosomes in pancreatic cancer cells, thereby down-regulating TGFBR3 expression and ultimately accelerating the development of PDAC via the activation of the TGF-β signaling pathway [88]. MiR-365 is enriched in EVs from M2 macrophages and can be transferred to PDAC cells, enhancing their proliferation, migration, and invasion potentials through the B-cell translocation gene 2 (BTG2)/focal adhesion kinase (F/ATP)-dependent tyrosine kinase (AKT) pathway, while inhibiting miR-365 in M2-EVs could repress malignant functions [89]. Moreover, M2-polarized TAMs can promote proteolytic activity and EMT of pancreatic cancer. Coculturing M2-polarized TAMs with pancreatic cancer cells resulted in high expression of Toll-like receptor 4 (TLR4) compared with culturing the M2-polarized TAMs alone. Small interfering RNA (siRNA)-mediated knockdown of TLR4 or inhibition of TLR4/IL-10 signaling with neutralizing antibodies could reverse the EMT of the pancreatic cancer cells, suggesting that M2-polarized TAMs can promote EMT and aggressive behavior in pancreatic cancer cells [90]. Selective packaging of different molecules into the TAM-EVs is involved in EV-mediated drug resistance. Recently, Binenbaum et al. reported that the presence of miR-365 in TAM-released EVs was found to be responsible for gemcitabine resistance in PDAC by upregulating the triphosphopyridine nucleotide (TPN) pool and inducing the enzyme cytidine deaminase. The latter can inactivate gemcitabine [91]. Xavier et al. found that chitinase 3-like-1 (CHI3L1) and fibronectin in the cargo of EVs shed by macrophages can also influence the pancreatic cancer cellular response to gemcitabine [92]. Overexpression of CHI3L1 and fibronectin induced PDAC resistance to gemcitabine through ERK activation. Inhibition of CHI3L1 and FN1 by pentoxifylline and pirfenidone, respectively, partially reverted drug resistance. In addition, M2 macrophage-derived EVs play important roles in the differentiation and activities of pancreatic cancer stem cells. MiR-21a-5p is upregulated in M2 macrophage-derived EVs and mediates the activities of pancreatic cancer stem cells via targeting krüppel-like Factor 3 (KLF3). Downregulating miR-21a-5p in M2 macrophage-derived EVs can inhibit Nanog/Oct4 expression and impair the sphere forming, colony-forming, invasion, migration, and anti-apoptosis abilities of pancreatic cancer stem cells [93]. The reprogramming of TAMs is a potential way to slow tumor progression in pancreatic cancer and a new avenue for improving its sensitivity to chemotherapy.

### Dendritic cells

Being the most potent and common type of antigen presenting cell, DCs express a wide range of TLRs and cytokines. Antigen presentation by DCs is critical for effective anti-tumor T cell responses. Activated DCs produce IL-12, TNF-α, and express major histocompatibility complex (MHC) class II molecules. However, DCs are inhibited in the pancreatic cancer microenvironment. Increased circulating levels of blood DCs have been associated with better survival in patients with PDAC [94, 95]. Interestingly, miR-203 is overexpressed in PDAC patient samples [96]. DCs downregulate TLR4, TNF-α, and IL-12 expression upon treatment with miR-203-rich exosomes derived from pancreatic cancer cells, which prevent DC-driven antigen presentation [97]. MiR-212-3p is also overexpressed in pancreatic cancer-derived exosomes, which make DCs unable to activate CD4^+^ T cells by inhibiting the expression of the regulatory factor X-associated protein (RFXAP) and consequently decreasing MHC II expression [98, 99]. This promotes the generation of an immunotolerant microenvironment. Chen et al. compared the expression profiles of normal DCs from healthy donors with DCs treated with pancreatic cancer-derived EVs. They identified 3227 and 924 differentially expressed long noncoding RNAs (lncRNAs) and mRNAs, respectively. LncRNAs such as ENST00000560647 and mRNAs such as lgmn possibly play critical roles in the immune escape of DCs treated with pancreatic cancer-derived EVs [100]. The fusion of tumor cells with DCs is an interesting method to improve tumor antigen presentation. Xiao et al. confirmed the immunogenicity of pancreatic cancer cells by response-induction via tumor EV-loaded DCs, which are well suited to present large amounts of pancreatic cancer-associated antigens. Moreover, the efficacy of vaccination immunotherapy with tumor EV-loaded DCs can be improved by combining it with drugs such as gemcitabine and/or sunitinib [101]. This approach results in a wide range of presented antigens, but clinical studies are necessary to evaluate its efficacy.

### Natural killer (NK) cells

NK cells belong to the innate lymphoid cell family and are involved in the cytotoxic killing of cancer cells. According to their CD56 levels, they can be classified as CD56^bright^ or CD56^dim^. The former group is dominant in secondary lymphoid tissues and plays an immunomodulatory role by producing high levels of cytokines, while the latter group represents the majority of NK cells in the blood, expresses high surface levels of CD16, and elicits a strong anti-cancer response [102]. NK cells that express different surface receptors can either promote or inhibit NK killing, depending on the overall prominence of activating versus inhibiting receptor signaling [103]. There is increasing evidence that NK cells can release EVs into the extracellular space to modulate tumor immunity [104]. NK cells can secrete EVs that contain typical NK markers (*e.g.,* CD56) and killer proteins (e.g., perforin, granzyme A & B, granulysin, and FasL) [105]. This release of cytotoxic proteins by activated human NK cells is the major mechanism for their cytotoxicity. Multiple cell killing mechanisms are activated by NK-derived EVs, including both caspase-independent and -dependent cell death pathways. Activation of caspase-3, -7, and -9 is detected in cancer cells incubated with NK-EVs, and caspase inhibitors block NK-EV-induced cytotoxicity, suggesting that NK-EVs can activate caspase pathways in target cells [106]. Wu et al. reported that the protein levels of cytotoxic proteins from NK-EVs isolates, including perforin, granzyme A, granzyme B, and granulysin, positively correlated with cytotoxicity. In addition, several endoplasmic reticulum-associated proteins are altered, suggesting that NK-EVs may induce endoplasmic reticulum stress that results in cell death [107]. EVs have good compatibility and can be explored as a drug carrier. Drug-loaded EVs effectively inhibit proliferation and induce apoptosis of tumor cells, thereby exerting anti-tumor effects. The NK cell-derived EV-entrapped paclitaxel (PTX-NK-EVs) enhance its anti-tumor effects by inducing the upregulation of Bax and caspase-3 in the apoptotic signaling pathway in tumor cells [108]. NK cells can also exert anti-tumor effects through EV-mediated delivery of nucleic acids. In pancreatic cancer cells, miR-3607-3p in NK-EVs suppresses cell migration and invasion through downregulation of IL-26, while a decrease in miR-3607-3p levels is associated with poor prognosis and tumor metastasis. IL-26 was found to be a direct target of miR-3607-3p in pancreatic cancer cells, which is highly expressed in pancreatic cancer tissues [109]. Tumor-derived EVs have the capability to suppress NK cell-mediated functions. Most of the inhibitory effects mediated by tumor-derived EVs on NK cell effector functions have been attributed to TGF-β1. TGF-β1 decreases the expression of NK cell activating receptors such as NKG2D, DNAM-1, NKp30, and NKp46, thus affecting NK cell recognition of cancer cells. Similarly, pancreatic cancer-derived EVs that express high levels of TGF-β1 strongly suppress NK cell functions and reduce their glucose uptake ability by inducing the phosphorylation of Smad2/3 [110, 111]. Thus, a better understanding of the mechanisms by which tumor-derived EVs influence the NK cell phenotype and functions can become new possibilities for cancer therapy. Potentiating NK cell activity or targeting NK cell-derived EVs could be encouraging strategies to be pursued in combination with anti-cancer treatments.

### Mesenchymal stem cells (MSCs)

In cancer, MSCs are a major component of the TME and play a key role in promoting tumor progression, trans-differentiating into MDSCs or M2-type macrophages and transforming the cellular milieu into one supportive of tumor survival [112]. MSCs release abundant amounts of EVs, which may be responsible for dissemination of messages from MSCs to recipient cells. The EVs produced by MSCs that are re-programmed by tumor-derived EVs can exert profound effects on tumor progression. Lung cancer cell-derived EVs can stimulate production and secretion of inflammatory cytokines, including IL-6, IL-8, and MCP-1, in MSCs via the NF-κB-TLR signaling pathway [113]. They can also increase secretion of IL-6 or IL-8 by recipient cells which, in turn, promotes cancer progression and EMT [114]. There is also evidence for anti-tumor activity of MSC-derived EVs. Bruno et al. found that EVs derived from BM-MSCs inhibited the growth and survival of various human tumor cell lines [115]. Other studies have confirmed the tumor-inhibitory potential of MSC-derived EVs in breast cancer or hepatocellular carcinoma cells, perhaps by upregulating immune effector cell functions [116, 117]. However, the mechanisms by which MSC-EVs inhibit tumor growth are still uncertain. It is reported that hsa-miR-143-3p was overexpressed in MSC-derived exosomes in pancreatic cancer. This miRNA can regulate KrasG12D, PI3K, ERK, JNK, p38MAPK, and vimentin synergistically to promote apoptosis and suppress cell growth, invasion, and migration. Moreover, lncRNAs MALAT1, SNHG1, and RP11-363N22.3 may also play critical roles in pancreatic cancer via hsa-miR-143-3p [118]. Yao et al. revealed that exosomal circ_0030167 derived from BM-MSCs could regulate miR-338-5p, enhance Wif1 expression, and inhibit the Wnt8/β-catenin pathway, thereby inhibiting the invasion, migration, proliferation, and stemness of pancreatic cancer cells [119]. Engineered MSCs represent a new potential therapeutic tool for improving the delivery of anti-cancer molecules in tumors. Besides the ability to engineer MSCs, MSCs can deliver drugs without genetic manipulation. Pascucci et al. showed that MSCs are able to package and deliver active drugs through their EVs with a higher cell-target specificity. EVs secreted by SR4987 cells primed with paclitaxel (SR4987PTX) successfully delivered active drugs and inhibited pancreatic cancer cell proliferation in a dose-dependent manner [120]. According to the study conducted by Kamerkar et al., electroporated MSC-derived exosomes containing an oncogenic Kras-targeting siRNA could suppress cancer in mouse pancreatic cancer models and remarkably improve overall survival [121]. MiR-124 in BM-MSC-derived exosomes suppressed the proliferation, invasion, migration, and EMT of pancreatic cancer cells, and also sensitized these cells to chemotherapy in an EZH2-dependent manner [122]. These results suggest a possible use of MSCs for the development of “biotech drugs” with enhanced anticancer efficacy and recruitment capacity.

## Potential applications of EVs in pancreatic cancer

The potential clinical applications of EVs in pancreatic cancer treatment are divided into the following categories: (1) early diagnostic biomarkers, (2) drug delivery, (3) chemoresistance targets, and (4) cancer immunotherapy.

### EVs as biomarkers

EVs can be isolated from certain patient cell types or body fluids such as blood, saliva, breast milk, cerebrospinal fluids, and malignant ascites. Because this is noninvasive, cancer-derived EVs have promising potential to be used as therapeutics and/or biomarkers. Hoshino et al. designed a panel of tumor type-specific EVP proteins, which can be used to classify tumors of unknown primary origin with 95% sensitivity and 90% specificity [123]. One study showed that exosomes derived from pancreatic cancer cells were enriched with a proteoglycan, glypican 1 (GPC1), on the surface of the parental cells. The presence of GPC1^+^ exosomes distinguished healthy subjects and patients with a benign pancreatic disease from patients with early-stage pancreatic cancer [124]. Following this observation, GPC1 in PDAC exosomes could be used as a highly specific biomarker for pancreatic cancer. Yang et al. reported that the sensitivity and specificity of diagnosis would be improved significantly when a panel of five markers (EGFR, EPCAM, MUC1, GPC1, and WNT2) were used for PDAC detection [125]. Combining diagnostic tools like GPC1-positive EVs, CA19-9, and EUS-FNA improves all diagnostic performance parameters and has an accuracy level as high as 84% [126]. GPC1^+^ EVs could also be used as a prognostic marker for patients with advanced pancreatic cancer receiving regional intra-arterial chemotherapy treatment. The proportion of GPC1^+^ EVs is higher in patients with advanced pancreatic cancer, which decreases following regional intra-arterial chemotherapy treatment. Furthermore, a greater decrease of GPC1^+^ EVs following regional intra-arterial chemotherapy is associated with improved survival rates of patients [127]. An effective EV isolation method is a technical challenge for the clinical application of EV biology because of their small size and high heterogeneity. Additionally, they are present in different biological fluids, including saliva, blood, plural effusion, and ascites [128]. EVs originating from different cell types in a biofluid vary in size, cargo content, and EV markers. At present, there is still no standardized method for their isolation. One of the major challenges in the field is finding a reliable, sensitive, and easily manipulated technique for specific EV populations isolation. Monguió-Tortajada et al. summarized and discussed the most used EV isolation methods [129]. Li et al. designed a multiplexed plasmonic immune-capture assay in combination with Surface-Enhanced Raman Scattering (SERS) nanotag technology. This chip, coupled with MIF GPC1 and EGFR antibodies, demonstrated the ability to detect PDAC-specific exosomes from as little as 2 μL of serum sample [130]. Liang et al. developed a rapid, ultrasensitive, and inexpensive nanoplasmon-enhanced scattering (nPES) assay that can directly quantify tumor-derived EVs from as little as 1 μL of plasma. The nPES assay for ephrin type-A receptor 2 (EphA2)-EVs, a pancreatic cancer EV biomarker, was able to distinguish pancreatic cancer patients from pancreatitis patients. EphA2-EVs could also predict the cancer stage and evaluate responses to neoadjuvant therapy better than a conventional enzyme-linked immunosorbent assay [131]. Alkaline phosphatase placental-like 2 (ALPPL2) is present in pancreatic cancer EVs and therefore has potential application in liquid biopsy-based diagnostic strategies. Shin et al. used the ALPPL2 binding aptamer to generate a diagnostic quantitative aptamer-linked immobilized sorbent assay (ALISA) for liquid biopsy. Direct ALPPL2 or CD9 antibody-based sandwich ALISAs were established, which could detect both free and EV-bound forms of ALPPL2 with high specificity and sensitivity [132]. Yokose et al. established an absolute quantification system for altered glycan-containing EVs elevated in pancreatic cancer patient serum. EVs recognized by O-glycan-binding lectins ABA or ACA were identified as candidate markers by lectin microarray. The ABA-or ACA-positive EVs were significantly increased in the serum of pancreatic cancer patients. These specific EVs with O-glycans can be used as potential biomarkers in a liquid biopsy for cancer screening [133]. Microbiome markers based on bacteria-derived EVs, which altered microbial compositions, are also candidate biomarkers for early diagnosis of pancreatic cancer. Among altered microbial communities, candidate biomarkers, such as *Verrucomicrobia* and *Actinobacteria* at the phylum and *Sphingomonas*, *Ruminococcaceae UCG-014*, *Propiobacterium*, *Akkermansia*, *Ruminiclostridium*, *Lachnospiraceae UCG-001*, and *Corynebacterium* at the genus level, from microbial EVs acquired from blood samples were identified for pancreatic cancer prediction [134]. Qin et al. developed an RNA-ratio based plasma samples which comprised eight EV-derived RNAs, including FBXO7, MORF4L1, DDX17, TALDO1, AHNAK, TUBA1B, CD44, and SETD3. This model could differentiate PDAC patients with a minimal AUC of 0.86. External validation using qRT-PCR data also exhibited a good classifier ability with an AUC of 0.89 when distinguishing PDAC from healthy controls [135]. Other potential biomarkers for PDAC include ZIP4, CD63, Rab5, MIF, and HULC from serum or plasma [136–139] (Summary in Table 2).

**Table 2:** Summary of EVs as diagnosis biomarkers in pancreatic cancer

Cargo	Sample	Methods for analysis of EVs	Findings	Diagnostic Performance	Refs.
GPC1	Serum	Flow analysis	The presence of GPC1^+^ exosomes distinguished healthy subjects and patients with a benign pancreatic disease from patients with early-stage pancreatic cancer	Sensitivity 100%; specificity 100%; AUC 1.0;	[124]
GPC1	Serum	Advanced multiplexed plasmonic assay	Sensitivity and specificity of diagnosis are improved significantly when a panel of five markers (EGFR, EPCAM, MUC1, GPC1 and WNT2) are used for PDAC detection	Sensitivity 86%; specificity 81%; diagnostic accuracy 84%	[125]
GPC1	Serum	Flow cytometry	Combining diagnostic tools, i.e. GPC1-positive EVs, CA19-9 and EUS-FNA improves all diagnostic performance parameters, and displays the best diagnosis accuracy as high as 84%	Sensitivity 64%; specificity 90%; Diagnosis accuracy 78%	[126]
GPC1	Plasma	Flow cytometry	GPC1 as a novel prognostic biomarker for patients with advanced pancreatic cancer following regional intra-arterial chemotherapy treatment	The level of GPC1^+^ EVs is associated with prognosis	[127]
EphA2	Plasma	nanoplasmon-enhanced scattering (nPES) assay	The nPES assay for ephrin type-A receptor 2 (EphA2)-EVs distinguishes pancreatic cancer patients from pancreatitis patients. EphA2-EVs are also predictive in cancer staging and in evaluating responses to neoadjuvant therapy	Sensitivity 94%;	[131]
ALPPL2	Cells	quantitative ALISA	Direct and ALPPL2 or CD9 anti-body-based sandwich ALISA were established, which could detect both free and EV-bound forms of ALPPL2	Not determined	[132]
O-glycan-binding lectins ABA or ACA	Serum	ExoCounter	EVs recognized by O-glycan-binding lectins ABA or ACA were identified as candidate markers by lectin microarray. The ABA-or ACA-positive EVs were significantly increased in the serum of pancreatic cancer patients	Sensitivity 7.8%; specificity 73.5%; AUC 0.838	[133]
Microbiome composition	blood samples	16S rRNA gene analysis	These microbiome markers, which altered microbial compositions, are candidate biomarkers for early diagnosis of pancreatic cancer with a high area under the receiver operating characteristic curve (0.966 and 1.000, at the phylum and genus level, respectively)	AUC 0.966;	[134]
EVs-derived RNAs	plasma samples	RT-qPCR	An RNA-ratio based plasma samples including eight EVs-derived RNAs, including FBXO7, MORF4L1, DDX17, TALDO1, AHNAK, TUBA1B, CD44, and SETD3	AUC 0.89	[135]
Zinc transporter protein ZIP4	Serum	proteomic analysis	The elevated serum levels of exosomal ZIP4 in patients with PDAC showed a diagnostic value of AUC 0.89	AUC 0.89	[136]
MIF	Plasma	ELISA	MIF was higher in exosomes from stage I PDAC patients who later developed liver metastasis and may be a prognostic marker for the development of PDAC liver metastasis	Not determined	[137]
CD63, Rab5	Serum	Western Blot	Alteration in the expression level in isolated exosomes of patients with pancreatic cancer was reported for proteins CD63 (3.17 fold) and Rab5 (1.73 fold)	Not determined	[138]
HULC	Serum	PCR	EVs-encapsulated HULC could be a potential circulating biomarker for early diagnosis PDAC	AUC 0.92	[139]

The combination of EV-based biomarkers is important to develop a molecular signature for diagnosing pancreatic cancer, as sensitive biomarkers specific to pancreatic cancer are essential for screening of asymptomatic individuals. Moreover, the efficiency and cost of the methodology for isolating and analyzing EVs need to be improved. A competitive cost, efficient testing, and validation in large clinical trials are needed for exploiting EV-based biomarkers for the diagnosis of PDAC [140].

### EVs as novel modes of drug delivery

Although numerous synthetic drug delivery systems have been developed,

applications of such systems are limited by inefficiency, cytotoxicity, and/or immunogenicity. EV characteristics such as small size, the presence of adhesive molecules on their surface, low toxicity, and reduced immunogenicity make them an optimal vehicle for drug delivery. Multiple cell types are used to obtain EVs, including tumor cells, immune cells, and mesenchymal cells [141]. EVs can be loaded with various therapeutic agents before or after the isolation process, including chemotherapeutics and nucleic acids. Transfection-based methods and co-incubation at 37 °C are the two well-known techniques for this goal. Other methods, including sonication, saponin-treatment, freeze–thaw cycles, and extrusion (passing EVs and substances through filters with decreasing pore sizes), have proven to be potential techniques for drug loading. Transfection is frequently used for transporting small RNAs into cells. These small RNAs can be introduced directly or via a vector into targeted cells. The co-incubation technique involves loading parental cells with molecules or chemotherapeutic compounds (e.g., paclitaxel). The originating cell-derived EVs will carry and transfer these cargo molecules to recipient cells [142]. Manipulating exosomes to facilitate drug delivery to PDAC tumors is an attractive strategy to increase drug bioavailability. Curcumin or paclitaxel can be loaded into EVs with this approach [120, 143, 144]. Incorporation of paclitaxel into exosomes from MSCs increases the cytotoxicity of this compound in cultured pancreatic cancer cells. Pascucci et al. loaded MSCs with paclitaxel and used the resulting EVs in pancreatic tumor cells, achieving an IC50 reduction of 2.54 ng/mL with the administration of free paclitaxel, and of 1.25 ng/mL when administered in EVs [120]. Another study also demonstrated the greater effectiveness of EVs derived from MSCs pre-treated with paclitaxel against pancreatic cancer cells, showing a significant proliferation inhibition and direct anti-cancer activity [145]. Drug-loaded EV preparations from different cell types exhibit distinctive loading capabilities and yield anti-tumor efficacies. Macrophage-derived EV-doxorubicin preparations can induce greater levels of apoptosis and higher anti-tumor activity in cancer cells compared with those derived from pancreatic cancer cells or PSCs [146]. Besides doxorubicin and paclitaxel, other drugs have also been explored. EVs loaded with gemcitabine reduced cell viability in pancreatic cancer cells by more than 50% compared with the free drug, and gemcitabine-loaded EVs achieved a significantly higher reduction in tumor size compared with free gemcitabine in murine models of pancreatic cancer [147].

Currently, some clinical trials have demonstrated the promising application of EVs in the clinic. A phase I study performed in the USA aims to use MSC-derived EVs loaded with a KrasG12D-specific siRNA to inhibit pancreatic cancer (http://clinicaltrials.gov; Clinical trial ID: NCT03608631). The clinical trial results are expected to be presented by March 2022. As novel modes of drug delivery, EVs are prospective for cancer treatment (Table 3). However, their functionality and physiological roles are still under investigation. Several issues, such as the purification, loading, targeting, and scaling-up of EVs, must be solved to transition the EVs from bench to bedside [148].

**Table 3:** Summary of studies investigating EVs as drug delivery and the therapeutic targets in pancreatic cancer

EVs source	Cargos	Findings	Refs.
MSCs	Paclitaxel	MSCs package and deliver active drugs through their MVs with a higher cell-target specificity and anticancer cytotoxicity	[120]
MSCs	Paclitaxel	EVs derived from MSCs pre-treated with paclitaxel against pancreatic cancer cells, showing a significant proliferation inhibition and direct anticancer activity	[145]
Macrophages	Doxorubicin	Macrophages-derived EVs-doxorubicin preparation induces greater apoptosis and higher antitumor activity in cancer cells compared to those derived from pancreatic cancer or PSCs	[146]
PDAC	Gemcitabine	Autologous exosomes facilitate cellular uptake of GEM and contributed to increased cytotoxic effect of GEM. Autologous exosomes also show targeting ability to pancreatic cancer in biodistribution study	[147]
MSCs	KrasG12D siRNA	The are expected to be presented by March 2022	NCT03608631
CAFs	miR-146a	The EVs of CAFs exposed to gemcitabine results in increasing expression of Snail (SNAI1) as well as the Snail target, microRNA-146a, which promote pancreatic cancer cells drug resistance	[67]
CAFs	miR-106b	MiR-106b can be directly transferred from CAFs to pancreatic cancer cells through EVs, which promotes gemcitabine resistance of cancer cells by targeting TP53INP1	[68]
PDAC	miR-155	Pancreatic cancer cells secrete EVs containing miR-155 to impact tumor-adjacent normal fibroblasts to convert them into CAFs, thus increasing drug resistance	[72]
Macrophage	miR-365	Transfer of miR-365 in macrophage-derived EVs induces resistance of pancreatic cancer cells to gemcitabine	[91]
PDAC	EphA2	EVs derived from chemo-resistant pancreatic cells confer gemcitabine resistance to sensitive cells via an EphA2-dependent mechanism	[151]

### Chemoresistance

EV-induced chemoresistance has been recognized as a novel mechanism of drug resistance. The bidirectional EV-mediated transfer of cargo to and from non-tumor cells significantly influences their response to anti-tumor treatments. CAF-derived EVs containing Snail, miR-146a, miR-106b, and miR-155 that are intrinsically resistant to gemcitabine can reportedly promote pancreatic cancer chemoresistance [67, 68, 72]. Gene expression analyses show the upregulation of ROS detoxification enzymes, such as catalase (CAT) and superoxide dismutase 2 (SOD2), and downregulation of deoxycytidine kinase in gemcitabine-exosome-treated cells. Deoxycytidine kinase is a gemcitabine-metabolizing gene, and downregulation occurs through exosomal miR-155. Either miR-155 suppression or deoxycytidine kinase restoration can lead to abrogation of pancreatic cancer chemoresistance mediated by EVs [149]. Pancreatic cancer EVs can assist chemoresistant tumor cells to transfer resistance to sensitive cells within the same tumor or at other anatomical sites [150]. Fan et al. reported that exosomes from chemoresistant PANC-1 cells increased the gemcitabine resistance of MIA PaCa-2 and BxPC-3 cells via an EphA2-dependent mechanism [151]. Modulating the production of EVs by blocking their secretion is a major avenue to mitigate the role of EVs in transferring drug resistance. Muralidharan-Chari et al. showed that inhibiting EVs release by preventing the activation of ERK using an inhibitor resulted in an increased sensitivity of pancreatic cancer cell lines to gemcitabine [152]. Thus, EVs from pancreatic cancer can promote chemoresistance by regulating RNAs, proteins, relevant genes, and signaling pathways [153]. In summary, EVs from pancreatic cancer or tumor microenvironments can promote chemoresistance. More studies are required to further explain how EVs mediate and promote related chemoresistance in pancreatic cancer.

### EV-based immunotherapy

Tumor-derived EVs contain immunosuppressive molecules such as PD-L1, TGFβ1, FasL, TRAIL, and NKG2D ligands, which make them important mediators of tumor immune evasion [154]. Basso et al. showed that conditioned medium from PDAC cells expressing SMAD4 induced an increase in the proliferation of T _Reg_ cells while decreasing that of CD8^+^ T cells [155]. By contrast, other studies showed that Hsp70-positive pancreatic cancer cells secrete exosomes containing high levels of Hsp70/Bag-4, which enhance the migration and cytolytic activity of NK cells towards Hsp70-positive cancer cells. This demonstrates that EV-derived signals can act to suppress or promote immune responses in cancer [156]. Besides immunosuppressive role of tumor-derived EVs that could be blocked for better immunotherapy outcomes, EVs could be used to activate the immune system because they share tumor antigens with their parental cells. These EVs not only induce potent anti-tumor effects mediated by CD8^+^ T cells, but also stimulate NK cell activity. EVs released by DCs express MHC class I and II molecules on their surface, along with T‐cell co‐stimulatory molecules, which are able to activate cognate T cells and induce immune responses. These activities motivate the use of DC-derived EVs in the treatment of cancer. Zitvogel et al. demonstrated that exosomes isolated from DCs exposed to tumor peptide were effective in inducing a tumor-directed immune response [157]. However, the clinical application of EV-based cancer immunotherapy still needs biochemical characterization research and extensive analysis of the underlying mechanisms.

## Future directions

The currently available studies reveal that there is a bidirectional transfer of molecules between pancreatic cancer cells and the stromal cells in TME. Inhibition of EVs-mediated intercellular communication may be an effective strategy to improve the response to treatment in PDAC patients. Moreover, EV-based clinical applications, such as diagnostic or prognostic biomarkers, drug carriers, chemoresistance targets, and cancer immunotherapy, have shown great promise. However, the current understanding of EVs is still incomplete and many challenges remain. First, since EVs are heterogeneous particles, it is difficult to isolate large quantities of pure and specific EVs from mixtures of different vesicle types. This is because of persistent technical challenges and a lack of suitable biomarkers for particular EVs. It requires not only a unified procedure of production, isolation, and characterization, but also a recognized criterion system for its safety and efficacy, as well as guidelines for the specific regimen of clinical application [158]. Second, it is important to explore which component of EVs is responsible for tissue-specific targeting, local environment modification, and immune alteration, in addition to further investigation into the underlying mechanisms. Third, exploiting the potential development of EVs as drug vehicles for effective therapeutic strategies can potentially improve cancer diagnosis and therapy. However, there are several issues that should be addressed before EVs can be used for clinical practice. Standard guidelines remain to be established for the manufacturing, purification, dosage, and duration of EV-based drugs [159]. Interactions between therapeutic EVs and unexpected cells should be avoided.

## Conclusions

Taken together, EVs play multifaceted roles in promoting pancreatic cancer, conferring therapeutic resistance, and remodeling the TME. There is a bidirectional transfer of molecules between pancreatic cancer cells and the stromal cells in TME. EV-based diagnosis and therapeutics in pancreatic cancer have shown great promise. EVs with specific cargo molecules have desirable diagnostic value and potential capability of predicting prognosis for pancreatic cancer patients. However, the clinical translation of EVs in cancer therapy still requires efforts considering many challenges, and each breakthrough will promote the clinical translation of EVs. With the significant increase in EV knowledge and the continuous development of biotechnology, EVs will have broader application prospects in pancreatic cancer diagnosis and treatment in the future.

## Data Availability

Not applicable, please refer to the original references.

## Abbreviations

ARF6: ADP-ribosylation factor 6
ALPPL2: Alkaline phosphatase placental-like 2
BTG2: B-cell translocation gene 2
CAFs: Cancer associated fibroblasts
CCL: C–C motif chemokine ligand
CHI3L1: Chitinase 3-like-1
DCs: Dendritic cells
EVs: Extracellular vesicles
ERK: Extracellular signal-regulated kinase
ESCRT: Endosomal sorting complex required for transport
EphA2: Ephrin type-A receptor 2
FGF2: Fibroblast growth factor 2
FAP: Fibroblast activation protein
FSP-1: Fibroblast- specific protein-1
Gemcitabine: GEM
GM-CSF: Granulocyte–macrophage colony-stimulating factor
KLF3: Krüppel-like Factor 3
MLCK: Myosin light chain kinase
MSCs: Mesenchymal stem cells
MHC: Major histocompatibility complex
mPGES-1: Microsomal prostaglandin E synthase-1
MDSC: Myeloid-derived suppressor cells
MCT-4: Monocarboxylate transporter-4
NK: Natural killer cells
nPES: Nanoplasmon-enhanced scattering
PDAC: Pancreatic ductal adenocarcinoma
PSCs: Pancreatic stellate cells
PLD: Phospholipase D
PDGF: Platelet-derived growth factor
ROS: Reactive oxygen species
RFXAP: Regulatory factor X-associated protein
SHH: Sonic hedgehog
SDF-1α: Stromal cell-derived factor-1α
TME: Tumor microenvironment
TAMs: Tumor-associated macrophages
TGF-β: Transforming growth factor-β
TCA: Tricarboxylic acid
EGF: Epidermal growth factor
TPN: Triphosphopyridine nucleotide
TLRs: Toll-like receptors
TNF-α: Tumor necrosis factor-α
5-LOX: 5-Lipoxygenase
